# Inhibition of GDF8 (Myostatin) accelerates bone regeneration in diabetes mellitus type 2

**DOI:** 10.1038/s41598-017-10404-z

**Published:** 2017-08-29

**Authors:** Christoph Wallner, Henriette Jaurich, Johannes Maximilian Wagner, Mustafa Becerikli, Kamran Harati, Mehran Dadras, Marcus Lehnhardt, Björn Behr

**Affiliations:** Department of Plastic Surgery, BG University Hospital Bergmannsheil, Ruhr University Bochum, Bürkle-de-la-Camp Platz 1, 44789 Bochum, Germany

## Abstract

Metabolic diseases like diabetes mellitus cause bone healing deficiencies. We found significant impairment of bone regeneration, osteogenic differentiation and proliferation in diabetic bone. Moreover recent studies suggest a highly underestimated importance of GDF8 (Myostatin) in bone metabolism. Our goal was to analyze the role of GDF8 as a regulator of osteogenic differentiation, proliferation and bone regeneration. We used a murine tibial defect model in diabetic (Lepr^db−/−^) mice. Myostatin-Inhibitor Follistatin was administered in tibial bony defects of diabetic mice. By means of histology, immunohistochemistry and QRT-PC osteogenesis, differentiation and proliferation were analyzed. Application of Myostatin-inhibitor showed a significant improvement in diabetic bone regeneration compared to the control group (6.5 fold, p < 0.001). Immunohistochemistry revealed a significantly higher proliferation (7.7 fold, p = 0.009), osteogenic differentiation (Runx-2: 3.7 fold, p = 0.011, ALP: 9.3 fold, p < 0.001) and calcification (4.9 fold, p = 0.024) in Follistatin treated diabetic animals. Therapeutical application of Follistatin, known for the importance in muscle diseases, plays an important role in bone metabolism. Diabetic bone revealed an overexpression of the catabolic protein Myostatin. Antagonization of Myostatin in diabetic animals leads to a restoration of the impaired bone regeneration and represents a promising therapeutic option.

## Introduction

Bone regeneration is usually an efficient process without scarring. However diseases such as diabetes mellitus can cause higher fracture rates accompanied by non-unions and a detrimentally diminished healing capacity^1–3^. In 2013, 382 million people were suffering from diabetes mellitus worldwide with a predicted increase to 592 million in 2035, thus diabetes appears to be the most common and epidemiologically most relevant endocrinological disease^4^. Summarized, the economic impact of diabetes associated bone healing impairment and current insufficient therapeutics lead to a high demand in developing novel treatment strategies.

Most studies, analyzing diabetes associated impairment of bone healing were generated in diabetes mellitus type 1 animal models. Expression of runt-related transcription factor-2 (Runx-2), a master gene of osteogenesis was shown to be significantly decreased in an intramembranous bone healing type 1 diabetes mellitus model. Simultaneously, a deficiency of osteoprogenitor cells in diabetes type 1 was revealed^5, 6^. The reasons for this are largely unknown, however, it was hypothesized that the expression of crucial growth factors is decreased by systemic diabetes mellitus^7–9^. Local substitution of Fibroblast Growth Factor 9 (FGF-9) and Vascular Endothelial Growth Factor (VEGF) in a diabetic murine unicortical tibial defect model revealed a significant enhancement of bone regeneration, angiogenesis, proliferation and osteogenic differentiation compared to diabetic control animals^10^. Nevertheless, exploring mechanisms and therapeutic options in the restricted diabetic bone regeneration requires continued efforts.

Myostatin (GDF-8), a member of the transforming growth factor-β superfamily, is a negative regulator of muscle growth. Myostatin-knockout mice (Myo^−/−^) show a significantly increased muscle mass^11, 12^. Furthermore Myostatin-knockout mice revealed an increased fracture callus, increased SRY-related HMG-box 5 (Sox-5) and Bone Morphogenic Protein (BMP-2) expression as a marker for early bone regeneration^13^. Although activation of Myostatin is reciprocally linked to a decrease of PCNA - a causal relationship is still not fathomed^14^.

Hamrick *et al*. showed that inhibition of Myostatin in the osteogenic differentiation of bone marrow stem cells is of great importance and consequently expression of osteogenic growth factors BMP-2 and Insulin-like growth factor 1 (IGF-1) increased^15^. In addition, inhibition of Myostatin led to an increase of osteogenesis and reduction of adipogenesis. Myo^−/−^ mice showed less body fat, lowered blood glucose levels and a general increase in density, strength and bone mineralization^16, 17^. Systemic effects of Myostatin knockout on parameters of the metabolic syndrome such as reduced blood glucose-, insulin-, and triglyceride values and a normalization of hyperphagia were confirmed in an additional study^11^.

Various models for the treatment of myositis and myopathy by application of Myostatin inhibition are currently undergoing intensive tests. Though Follistatin is not very selective and putatively inhibits GDF11 and Activin A, it is one of best available most cost-effective inhibitors of Myostatin^18–22^. It has been shown despite of a blockade of Myostatin target receptor ActRII (Activin Receptor 2) Follistatin also directly binds to Myostatin transforming it into an inactive form^23^. This has been shown *in vivo* ^18^. For instance, the potent Myostatin inhibitor Follistatin affecting the Myostatin pathway has been shown to be clinically successful in treating muscular diseases. Additionally, transfection of Follistatin gene in human Phase 1/2a studies is a promising example of gene therapy for treating myopathies. However, these studies are exclusively limited to muscular diseases^20, 24–26^.

Thus, inhibition of Myostatin increases the osteogenic potential and bone mineralization, and reduces the impact of the metabolic syndrome. However, its utilization in increasing bone regeneration in general and in the diabetic condition in particular has not yet been investigated. The goal of this application is to use Myostatin inhibitors as a therapeutic approach to improve diabetic bone regeneration. Upon local application of Follistatin we aim to successfully block the Myostatin pathway and subsequently improve bone regeneration, osteogenic differentiation and proliferation in a murine diabetic model.

## Material and Methods

### Animal surgery

All animal experiments were approved by the IACUC LANUV NRW (The Ministry for Environment, Agriculture, Conservation and Consumer Protection of the State of North Rhine-Westphalia; 84–02.04.2016.A045). All methods were performed in accordance with the relevant guidelines and regulation by the IACUC LANUV NRW. Heterozygous *Lepr* ^*db*^ (*db* ^+^ */db* ^−^) mice were obtained from Jackson Laboratory (#000697) and kept with unlimited access to water and standard laboratory chow. Heterozygous *db* ^+^ */db* ^*−*^ mice on a C57BL/6 J background were mated to obtain WT, *db* ^+^ */d−* ^*-*^ and *db* ^*−*^ */db* ^*−*^ mice. Genotyping for breeding was performed on genomic DNA by restriction enzyme digest after PCR (forward primer: ATGACCACTACAGATGAACCCAGTCTAC; reverse primer: CATTCAAACCATAGTTTAGGTTTGTC) according to Horvat and Bügner^27^. Female littermates at the age of 16 to 20 weeks were used for all experiments. All surgical procedures were performed under inhalation anesthesia with isoflurane and buprenorphine (Abbott GmbH). An established murine tibial defect model was performed as previously described^9, 10^. Briefly, after shaving and disinfecting the left leg, an incision was made on the proximal anterior skin surface over the tibia. After splitting the anterior tibial muscle, the tibia was properly exposed. A 1 mm unicortical defect was created on the anterior tibial surface (approx. 3 mm under the knee joint) and filled with a 1 mm circular collagen sponge coated with a reagent according to the animal group. The 3 animal groups included (1) WT control mice with PBS, (2) diabetic control animals with PBS, (3) diabetic animals treated with 2 µg Follistatin. Wound closure was performed with 6–0 Prolene interrupted sutures. Each group consisted of at least 7 animals. Euthanasia was performed according to national and international laws and guidelines. Briefly, cervical dislocation was performed after thorough anesthesia to harvest tissue.

### Isolation of mouse adipose-derived stem cells (mASCs)

mASCs were harvested from inguinal fat pads of Lepr^db−/−^ Black 6 mice and Black 6 WT mice and labeled similarly as previously described^28^. Inguinal fat pads were excised and washed in betadine/PBS. Thereafter, fat pads were minced under sterile conditions and digested with 0.1% Collagenase A (Roche Diagnostics, Mannheim) for 30 min at 37 °C in a shaking water bath, centrifuged down, washed and plated. Culture conditions were performed with DMEM GlutaMAX, 10% FBS and 100 IU/ml penicillin/ 100 IU/ml streptomycin for *in vitro* experiments. At passage 1 and 80% confluence, osteogenic differentiation was initiated. Osteogenic differentiation medium (additional β-Glycerolphosphate 1%, ascorbic acid 0.1% modificated from Neuhuber *et al*.^29^) was changed every third day with glucose concentration accordingly. Cells were incubated at 37 °C with a carbon dioxide level at 5%.

Neutralizing (ND_50_) antibodies for Activin (7.5 µg/ml - Goat, polyclonal, R&D Systems, AF338) and Myostatin (3 µg/ml – Goat, polyclonal, R&D Systems, AF788) were used as described before^30^.

### Quantification of osteoid formation

Tibiae were harvested at a given time and fixed in 4% paraformaldehyde (Sigma Aldrich) overnight at 4 °C and decalcified in 19% EDTA (Applichem) for five days with daily changes of the solution. Samples were then dehydrated and embedded in paraffin and cut into serial sagittal sections (thickness 6 µm).

Every 6^th^ section was used to characterize bone formation with aniline blue (Roth) staining as previously described^31^. Images were taken with a bright field microscope (Zeiss Axiovert 100, Zeiss) and following settings with Axiovision 4.8: objective 2.5x, exposure time 614 ms, dimensions 3900 × 3090 Px, scanned color. Histomorphometric measurements of aniline blue stained sections were performed in Adobe Photoshop with modifications^9^. Additionally, Goldner stainings were performed according the standard protocol^32^. Briefly, a 2000 × 2000 Px dimensioned selection box was placed to cover the entire defect area. Utilizing the Adobe Magic Wand Tool (settings: tolerance 60%; no-contiguous) new osteoid formation was selected semi-automatically. Tonal separation in two steps and deselecting existing cortical bone resulted in highlighted pixels reliably corresponding to bone formation area.

Trabecular Area (Tb.Area) and Osteoblast Surface (Ob.S) were calculated following the 2012 published recommendations of the American Society for Bone and Mineral Research^33^. Osteoid Area per Total Area (OA/TA) was measured as described before^34^.

Alizarin Red staining (28 days after osteogenic differentiation) was performed as previously described^28, 35^.

### Immunohistochemistry

For immunofluorescence stainings of RUNX-2 (rabbit, polycloncal, SantaCruz Biotechnology, sc-10758, 1:50, Runx2 (M-70)), Activin A (Goat, polyclonal, R&D Systems, AF338, 1:100, Activin A), PCNA (rabbit, polycloncal, SantaCruz Biotechnology, sc-7907, 1:100, PCNA (Fl-261)), PECAM-1 (rat, monoclonal, BD Pharmingen, 553370, 1:400, PECAM, AB_394816), ALP (mouse, polycloncal, SantaCruz Biotechnology, sc- 166261, 1:100, Alkaline Phosphatase), Myostatin (rabbit, polycloncal, SantaCruz Biotechnology, sc-6885-R, 1:100, GDF8), p-38 MAPK (rabbit, Cell Signaling, 4511 S, 1:800, p38 MAPK (T180/Y182)), GDF 11 (mouse, monocloncal, Santa Cruz Biotechnology, sc-393335, 1:100, GDF 11), SMAD 1 (mouse, monocloncal, Santa Cruzt Biotechnology, sc-7965, 1:100, SMAD 1) and SMAD 2/3 (rabbit, Cell Signaling, 8828, 1:200, SMAD2/3) sections were incubated at 58 °C for 1 h and subsequently rehydrated and incubated with 0.125% Proteinase K for 15 min at 37 °C. After a short washing step with PBS, sections and chamber slides were permeabilized with 0.1% Tween 20 for 4 min and treated with blocking solution for 1 h. Incubation with primary antibodies followed overnight in blocking solution at 4 °C. After washing with PBS, a rabbit or goat biotinylated secondary antibody conjugated with AlexaFluor488 or AlexaFluor594 was used for detection. All sections have been counterstained with DAPI. Sections were subsequently mounted with Fluoromount Aqueous Mounting Medium (Sigma Aldrich). Images for immunofluorescence were taken with a fluorescence microscope (Olympus IX3-Series).

Analogous to quantification of bone formation, a region of interest was selected (2,000 × 2,000 Px). Immunohistochemically positive stained pixels were automatically selected by using the Adobe Magic Wand Tool (settings: tolerance 60%; noncontiguous).

Osteocalcin was immunodetected with Osteocalcin antibody (rabbit, polycloncal, SantaCruz Biotechnology, sc-30045, 1:100, Osteocalcin, AB_653627). Similar to immunofluorescence sections were incubated with primary and secondary antibody. After washing with PBS, a rabbit biotinylated secondary antibody followed by the AB reagent and NovaRed (Vector Laboratories) was used for detection. Images were taken with an AxioImager M2 Imaging System (Zeiss). For histormorphometry of Osteocalcin Osteocalcin positive cells were counted in randomly detected boxes (100 µm × 100 µm) within the defect area.

### RNA preparation and cDNA synthesis

The homogenization of harvested bone was achieved with an in 1 ml Trizol reagent (Life Technologies, Darmstadt, Germany) on ice. Tissue war processed as described before^36^. Subsequently, RNA purification was done with RNeasy Mini Kit (Qiagen, Hilden, Germany) according to manufacturer’s instructions. Synthesis of cDNA was performed by means of the High Capacity cDNA Reverse Transcription Kit with RNase inhibitor (Life Technologies) using 200 ng total RNA per reaction.

### Quantitative real-time PCR

Quantitative determination of relative gene expression was performed on Applied Biosystems 7900HT Fast Real-Time PCR System (96 well plates) using TaqMan® gene expression assays (MSTN, Hs00976237_m1; ACVR1C, Hs00899854_m1) and TaqMan® universal master mix (Applied Biosystems, Darmstadt, Germany). For each reaction, 2 ng cDNA were used. Data were analyzed according to the manufacturer’s ΔΔCt method (Applied Biosystems). 18 S was used as a reference gene.

### Radiography

Standardized digital radiographs (exposure parameters: 40 kV, 0.5 mAs) of the operated tibiae were completed 28 days after surgery to detect mineralization of the defects. The methods was described before. To further determine the ossification additional luminance assays of the region of interest (ROI: 40 × 20 Px) were performed^37, 38^.

### Statistical analysis

Results of the study are presented as mean ± standard error of the mean (SEM) of at least three independent experiments. P-values were calculated by student’s t-test comparing two groups and ANOVA if comparing more than two groups. Statistical significances were set at a p-value < 0.05.

## Results

### Myostatin expression is significantly increased in diabetic bone tissue

**Figure 1:**
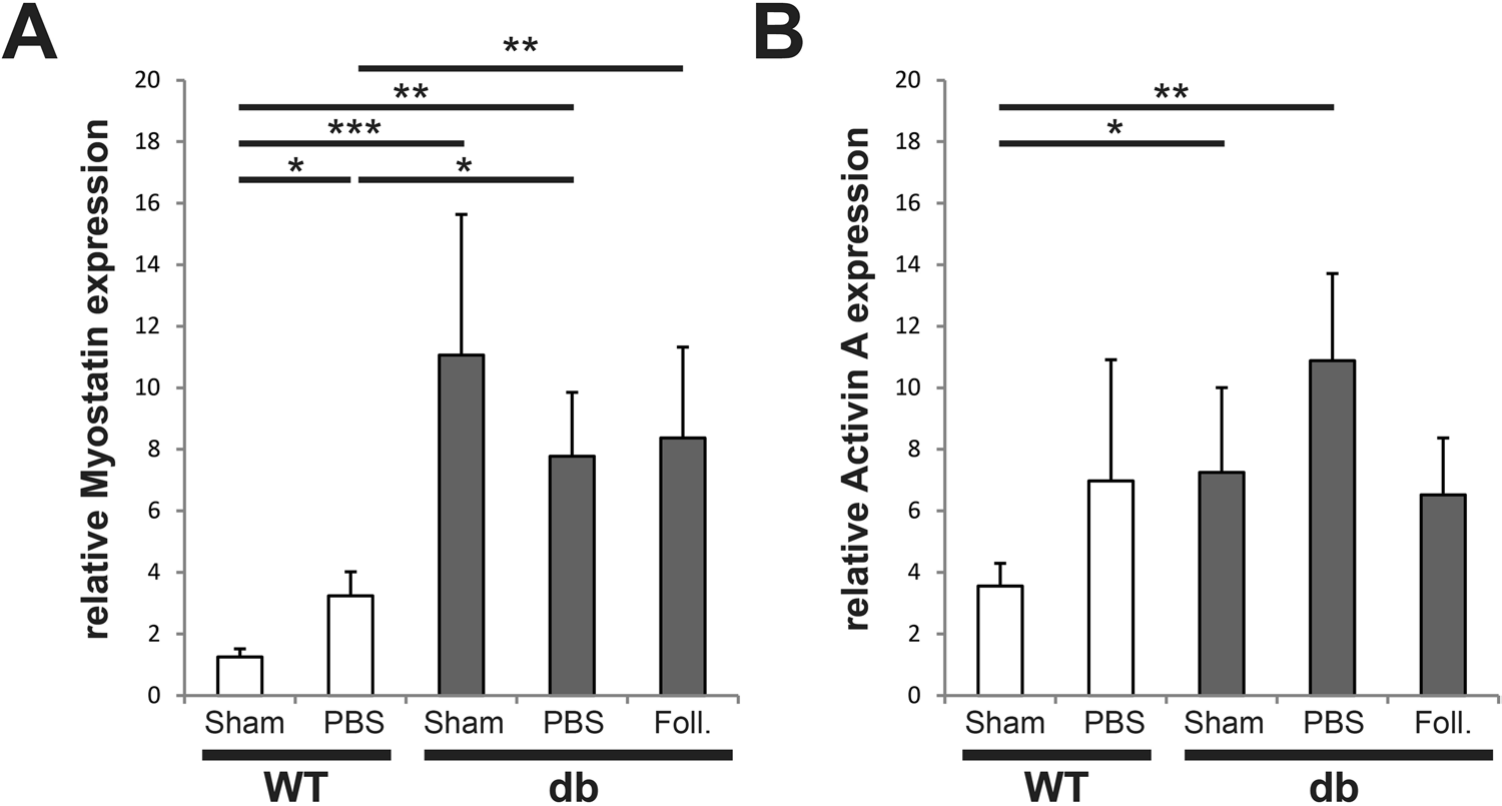
Expression of Myostatin and Activin A is highly upregulated in uninjured diabetic bone as compared to WT (shown in arbitrary units). (**A**) qPCR of uninjured (Sham) and injured postoperative (3dpo) mice showed increased expression of Myostatin in uninjured diabetic and injured diabetic and WT bone. Follistatin (Foll.) treated diabetic bone showed no significant difference compared to diabetic uninjured and injured bone. Follistatin treatment did not alter Myostatin expression compared to control animals (diabetic bone with PBS). (**B**) qPCR showed increased level of Activin A in diabetic bone compared to WT bone. Follistatin treatment did not alter Activin A expression compared to control animals (diabetic bone with PBS). Results are shown as means ± SEM. P-value: * < 0.05; ** < 0.01; ** < 0.001 (two sample t-test).

Unicortical defects were performed on diabetic and wildtype animals. First we analyzed Myostatin and Activin A expression in uninjured (Sham) and 3 days after setting a unicortical tibial defect (Fig. 1). A group with Follistatin treatment (2 µg Follistatin) was included to observe an inhibition of Myostatin and Activin A gene expression by Follistatin. A 9.4 fold increase of Myostatin expression was observed in uninjured diabetic bone (p < 0.0001), while a 2.1 fold increase of Activin A (p = 0.03) was detected. While injured bone has a 2 fold increase of Myostatin expression in wildtype animal (p = 0.018) there is a decline in diabetic injured bone by 34%. Placing a tibial defect lead to non-significant increase of Activin A both in wildtype and diabetic animals. Uninjured diabetic bone showed elevated Activin A levels compared to their wildtype correlate (p = 0.024).

### Inhibition of Myostatin by Follistatin enhances osteogenic differentiation and calcification *in vitro*

**Figure 2:**
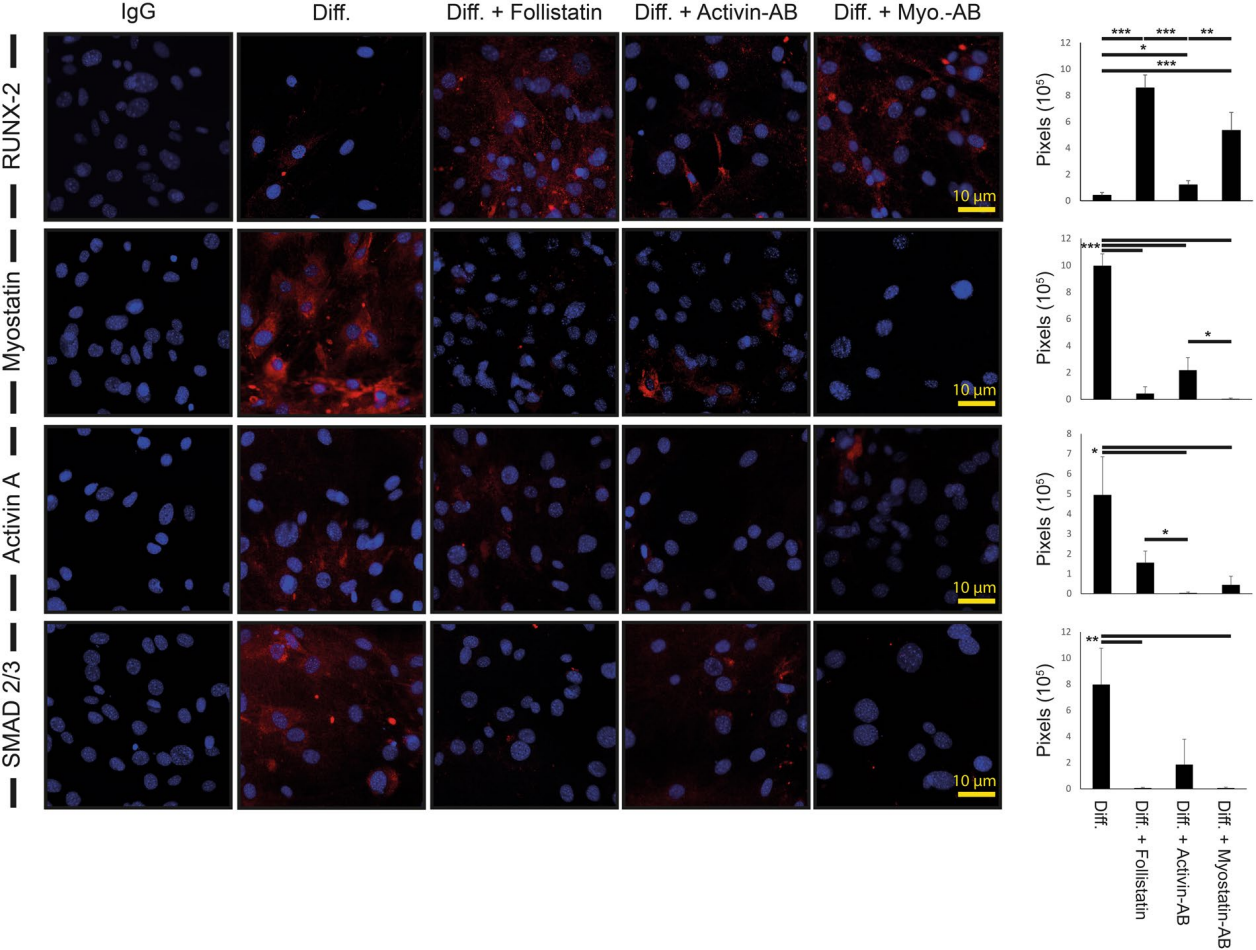
Reduced SMAD2/3 expression and increased RUNX-2 transcription is mediated by inhibition of Myostatin *in vitro*. **(Left)** mASC from Lepr^db−/−^ Black 6 mice were cultured for 7 days in osteogenic differentiation medium without or with either Follistatin Activin neutralizing Antibody (Activin-AB) or Myostatin neutralizing antibody (Myo.-AB). **(Right)** Quantification of immunofluorescence positive pixels for Runx-2 (red) showed increased Runx-2 levels in Follistatin and Myo.-AB treated groups compared to only differentiation medium. Myostatin positive pixels (red) were reduced in all treatment groups compared to the standard differentiation medium group. Significantly decreased Activin A positive levels were observed in the Activin-AB and Myo.-AB group but not in the Follistatin treated group compared to the standard differentiation medium group. SMAD 2/3 positive levels were reduced in the Follistatin and Myo.-AB group but not in the Activin-AB group compared to the standard differentiation medium group. Scale bar: 10 μm. Results are shown as means ± SEM. P-value: * < 0.05; ** < 0.01; *** < 0.001; (two sample t-test).

mASC of Lepr^db−/−^ Black 6 mice were differentiated in osteogenic medium as described before for 7 days to evaluate the pro-osteogenic effect of Follistatin application^35^. Differentiation medium combined with Follistatin or Myostatin neutralizing antibody (3 µg/ml) showed significantly enhanced osteogenic differentiation, while Activin neutralizing antibody (7.5 µg/ml) showed a less osteogenic differentiative effect. Myostatin was most significantly inhibited by Follistatin (23 fold decrease) and Myostatin neutralizing antibody (244 fold decrease) administration. Activin A levels were significantly reduced in Follistatin (3 fold decrease), Myostatin (11 fold decrease) and Activin A (147 fold decrease) neutralizing antibody treated groups. SMAD 2/3 level was significantly reduced by Follistatin (130 fold decrease) and Myostatin neutralizing antibody treatment (126 fold decrease) (Fig. 2).

Osteogenic differentiation of mASC of WT and diabetic origin for 28 days showed a decline of calcification in mASC of diabetic origin compared to WT mASC in the Alizarin red staining. This negative effect was eliminated upon Follistatin application (9 µg/ml).

### Unbounded Myostatin levels and Myostatin signaling are significantly decreased by local application of Follistatin

**Figure 3:**
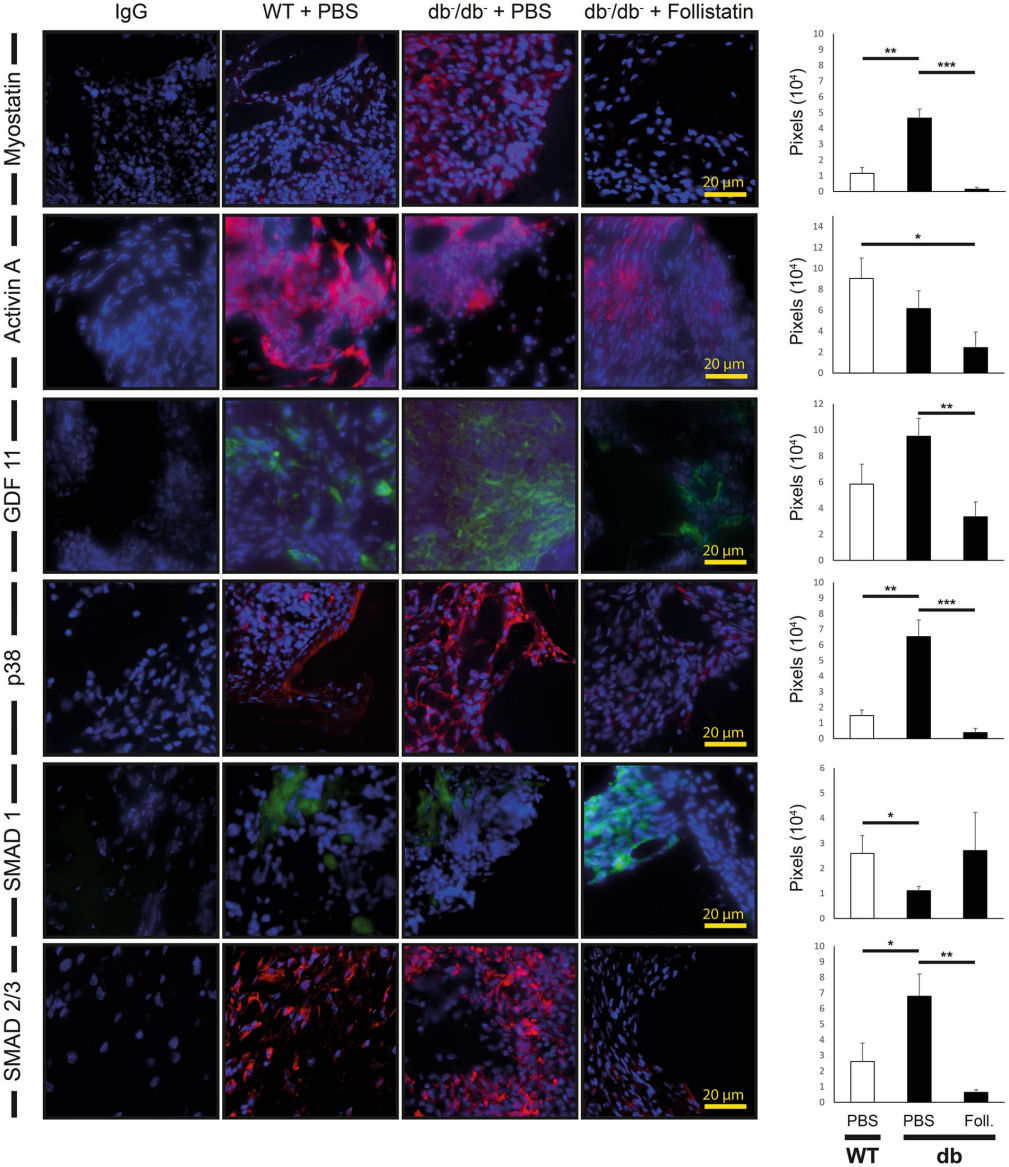
Local application of Follistatin inhibits Myostatin and GDF 11 and downstream targets *in vivo*. **(Left)** Myostatin (red), GDF 11 (green), Activin A (red), downstream targets and BMP 7 downstream target SMAD 1 (green) were detected by immunofluorescence for p38 (red) and SMAD 2/3 (red) (3 days postoperatively). (**Right)** Quantification of immunofluorescence positive pixels revealed that Follistatin treatment in diabetic mice significantly reduced Myostatin and GDF 11 levels and downstream targets p38 and SMAD 2/3 compared to elevated levels in diabetic animals without treatment. Blockage of Myostatin and downstream targets with Follistatin showed reduced levels of p38 and SMAD 2/3 compared to wildtype animals, while no difference in SMAD 1 levels was detected upon Follistatin treatment. Follistatin application does not significantly decrease Activin A levels in diabetic mice. Results are shown as means ± SEM. P-value: *** < 0.001, (two sample t-test). Scale bar: 20 μm. Foll. = Follistatin. Results are shown as means ± SEM. P-value: * < 0.05; ** < 0.01; *** < 0.001; (two sample t-test).

Next, we sought to investigate the effects of Myostatin inhibition on bone regeneration. In order to verify sufficient inhibition of Myostatin by local application of Follistatin into the defect, immunohistochemical stainings for Myostatin and subsequent downstream targets p38 and SMAD 2/3 were performed on day 3 postoperatively. Accordingly, the selectivity of Follistatin application was determined by immunofluorescence for Activin A, SMAD 1 (downstream target of BMP 7) and GDF 11. Immunohistochemistry revealed significantly increased levels of unbounded Myostatin (5 fold), p38 (4 fold) and SMAD 2/3 (2.5 fold) in diabetic tibial defects as compared to wildtype, while locally applied Follistatin in diabetic animals showed a significant decrease of all three proteins (unbounded Myostatin: 31 fold, p38: 17 fold, SMAD 2/3: 10.6 fold) compared to diabetic control animals. Activin A positive signal was decreased upon Follistatin treatment in diabetic animals in compared to WT but not compared to the diabetic control group. GDF 11 was not significantly increased in diabetic bone defects, however Follistatin application lead to a further decrease of GDF 11 in diabetic bone compared to diabetic defects (2.8 fold, p = 0.008). SMAD 1, the downstream target of BMP 7 was decreased in diabetic defects (2.3 fold, p = 0.04) but not significantly increased by Follistatin application (Fig. 3).

### Follistatin application increases proliferation and osteogenic differentiation

**Figure 4:**
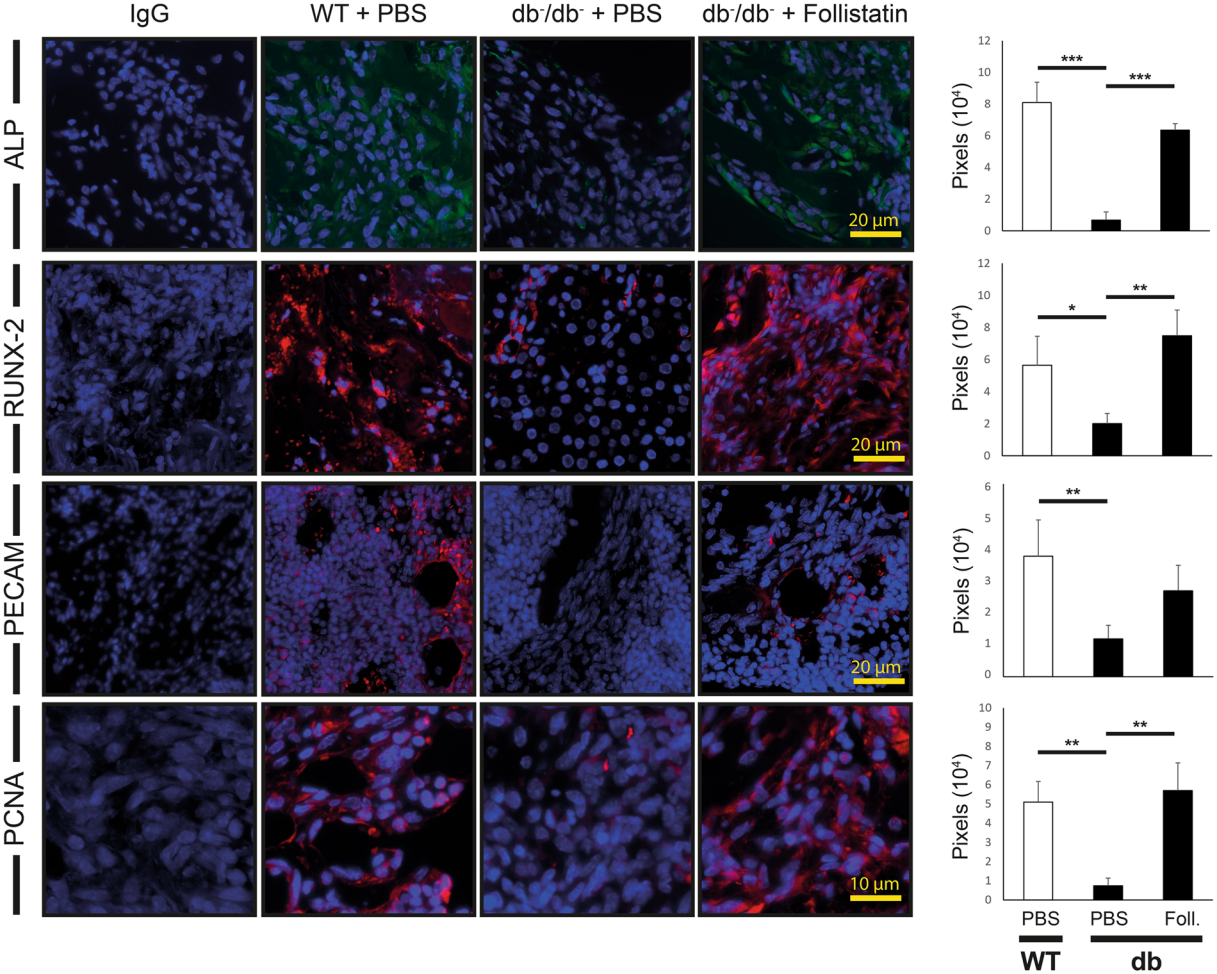
Follistatin enhanced osteogenic differentiation and proliferation. **(Left)** Osteogenic differentiation was detected by immunofluorescence for ALP (green) and RUNX-2 (red), proliferation by immunofluorescence for PCNA (red). **(Right)** Quantification of immunofluorescence positive pixels revealed that osteogenic differentiation is significantly increased in Follistatin treated diabetic defects compared to the control group db^−^/db^−^ and similar to WT mice ALP and RUNX-2 activity. Follistatin treatment in diabetic mice revealed a significant enhancement of proliferation compared to diabetic control animals and reached level of wildtype animals. Scale bar ALP + RUNX-2: 20 μm, PCNA: 10 μm. Foll. = Follistatin. Results are shown as means ± SEM. P-value: * < 0.05; ** < 0.01; *** < 0.001; (two sample t-test).

To evaluate the osteogenic and proliferative potential upon local Myostatin inhibition *in vivo*, immunohistochemistry for ALP, RUNX-2 (osteogenic differentiation), PECAM (vascular formation) PCNA (proliferation) was carried out 3 days postoperatively. ALP (12 fold, p < 0.001), RUNX-2 (2.8 fold, p = 0.054), PCNA (7 fold, p = 0.006) and PECAM (3.5, p = 0.03) were significantly reduced in diabetic control animals compared to wildtype animals (Fig. 4). In contrast local application of Follistatin into diabetic tibial defect significantly increased ALP (9.3 fold, p < 0.001), RUNX-2 (3.7 fold, p = 0.011) and PCNA (7.7 fold, p = 0.009) but not PECAM expression compared to diabetic animals (Fig. 4).

**Figure 5:**
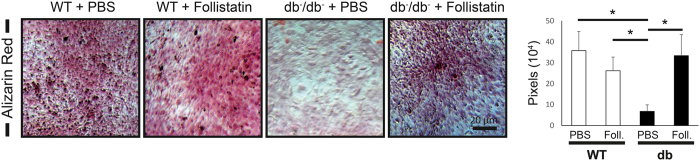
Follistatin enhances calcification of osteogenic differentiated mASC *in vitro*. Alizarin red staining of 28 days osteogenic differentiated mASCs showed increased mineralization in Follistatin treated cells compared to diabetic control. Scale bar: 20 μm. Results are shown as means ± SEM. P-value: * < 0.05; ** < 0.01; *** < 0.001; (two sample t-test).

28 days of osteogenic differentiation of WT and Lepr^db−/−^ mASC showed decreased calcification of Lepr^db−/−^ mASC (5.3 fold, p = 0.01) compared to WT mASC *in vitro*. The application of Follistatin (5 µg/ml) enhanced calcification in mASC of diabetic origin (4.9 fold, p = 0.024) significantly compared to the diabetic control (Fig. 5).

### Follistatin enhances osteoid regeneration

**Figure 6:**
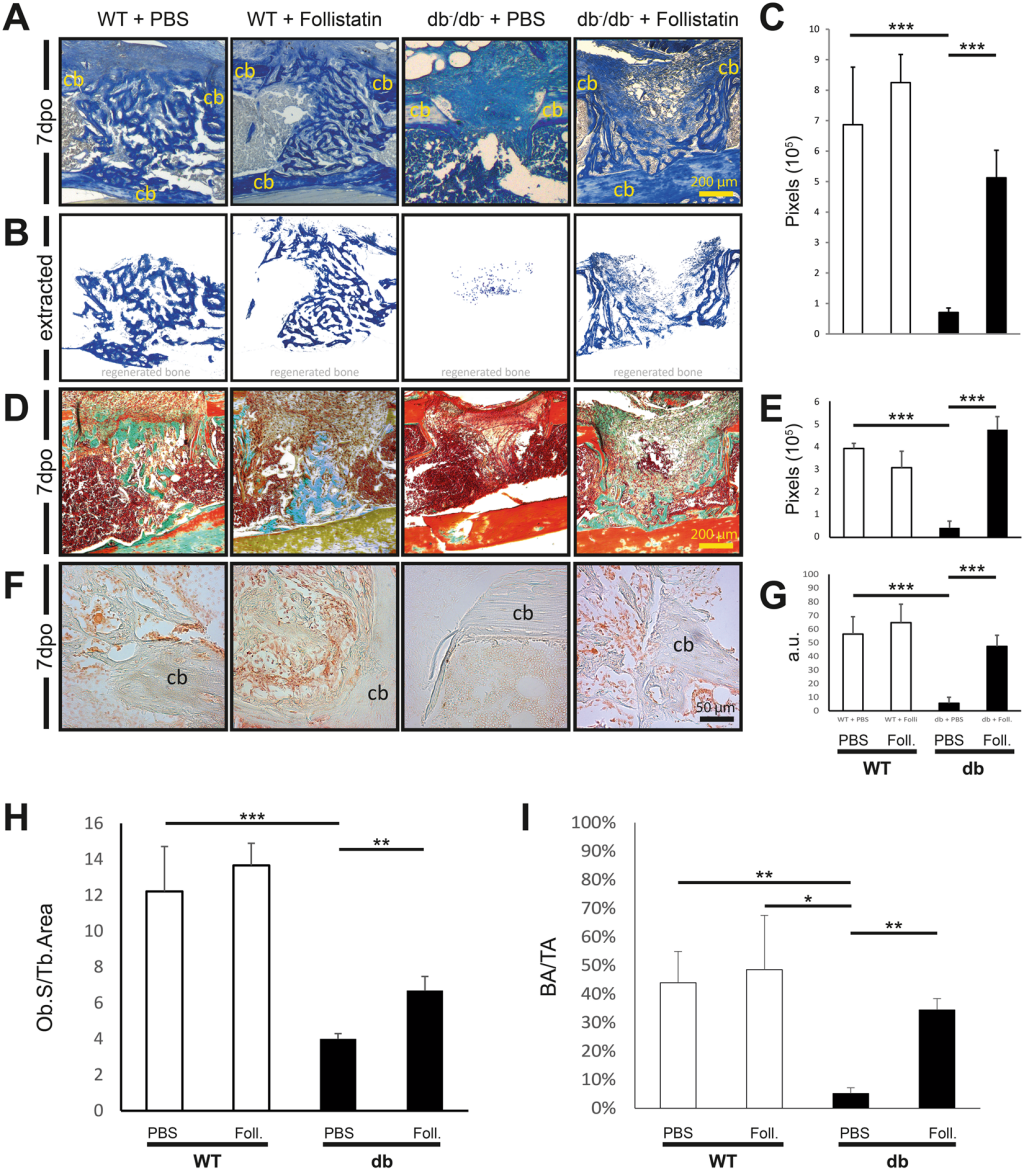
Follistatin enhanced diabetic bone regeneration – histology. **(A)** Aniline blue staining of db^−^/db^−^ control mice showed impaired new osteoid formation compared to WT control mice, while diabetic defects treated with Follistatin showed improved osteoid formation compared to diabetic control mice. WT mice treated with Follistatin showed a slight but not significant improvement. **(B)** Extracted osteoid formation area. **(C)** Quantification of aniline blue positive pixels (6 histological sections per sample) revealed that osteoid formation is significantly increased in Follistatin treated diabetic defects compared to the control group db^−^/db^−^ and similar to osteoid formation of WT mice 7dpo (7 days postoperatively). Follistatin treatment in WT mice did not showed significantly improved osteoid regeneration. **(D)** Goldner staining of db^−^/db^−^ control mice revealed impaired osteoid formation compared to WT, while diabetic defects treated with Follistatin showed improved osteoid formation. **(E)** Quantification of acid green positive pixels. **(F + G)** Immunohistochemistry for Osteocalcin demonstrated reduced Osteocalcin positive cells in diabetic animals on 7dpo, while local application of Follistatin restored the Osteocalcin positive cell number to the WT group level. **(H)** Calculation of Ob.S/Tb. Area showed diminished osteoblast number in db^−^/db^−^ compared to wildtype mice. The treatment with Follistatin enhanced the number significantly. **(I)** Calculation of OA/TA revealed reduced osteoid regeneration in diabetic animals compared to WT animals, while the application of Follistatin increases the regeneration compared to diabetic condition. Scale bar A + D: 200 μm. Scale bar F: 50 μm. Cb indicates cortical bone. dpo = days postoperatively. Ctrl = Control. Foll. = Follistatin. Results are shown as means ± SEM. P-value: * < 0.05; ** < 0.01; *** < 0.001; (two sample t-test).

To analyze osteoid formation after local application of Follistatin *in vivo*, aniline blue and Goldner-staining were utilized to detect osteoid formation 7 days postoperatively. Histomorphometry of the stainings revealed a significant decrease of osteoid regeneration in diabetic animals compared to WT animals (Fig. 6A–D, H/I). Aniline Blue staining of diabetic defects demonstrated 8.2 fold decrease in osteoid area (p < 0.001) compared to WT animals, while local application of Follistatin (2 µg) rescued osteoid formation (6.5 fold, p < 0.001). Follistatin treatment of wildtype animals did show a slight, but not significant improvement in osteoid formation compared to wildtype control animals (Fig. 6A–D). To further validate bone regeneration quality a Golder staining was performed. Again, diabetic defects showed a 9.5 fold decrease in osteoid area compared to WT (p < 0.001). The administration of Follistatin locally in the defect enhanced diabetic osteoid formation by 11 fold (p < 0.001) compared to control diabetic animals (Fig. 6D/E).

Immunohistochemistry for Osteocalcin (Osteocalcin positive cells per 10.000 µm²) confirmed a decreased osteogenic differentiation in diabetic defects (7 dpo) compared to WT (10 fold decrease, p > 0.001), while the application of local Follistatin to diabetic defects rescued the impaired osteogenic differentiation (8.5 fold increase, p > 0.001) (Fig. 6F/G).

Calculation of osteoblast surface per trabecular osteoid area showed decreased osteoblast count in diabetic animals (3 fold, p < 0.001), while Follistatin treated defect showed an increase in osteoblast count (1.7 fold, p = 0,008) compared to diabetic bone without treatment (Fig. 6H). Evaluation of osteoid area per tissue area confirmed these findings. The OA/TA (osteoid area per tissue area) in the diabetic defect area was reduced 8.8 fold (p = 0.005) compared to WT bone. Follistatin treated defects showed a 6.8 fold increase of BA/TA compared to diabetic controls (Fig. 6I).

**Figure 7:**
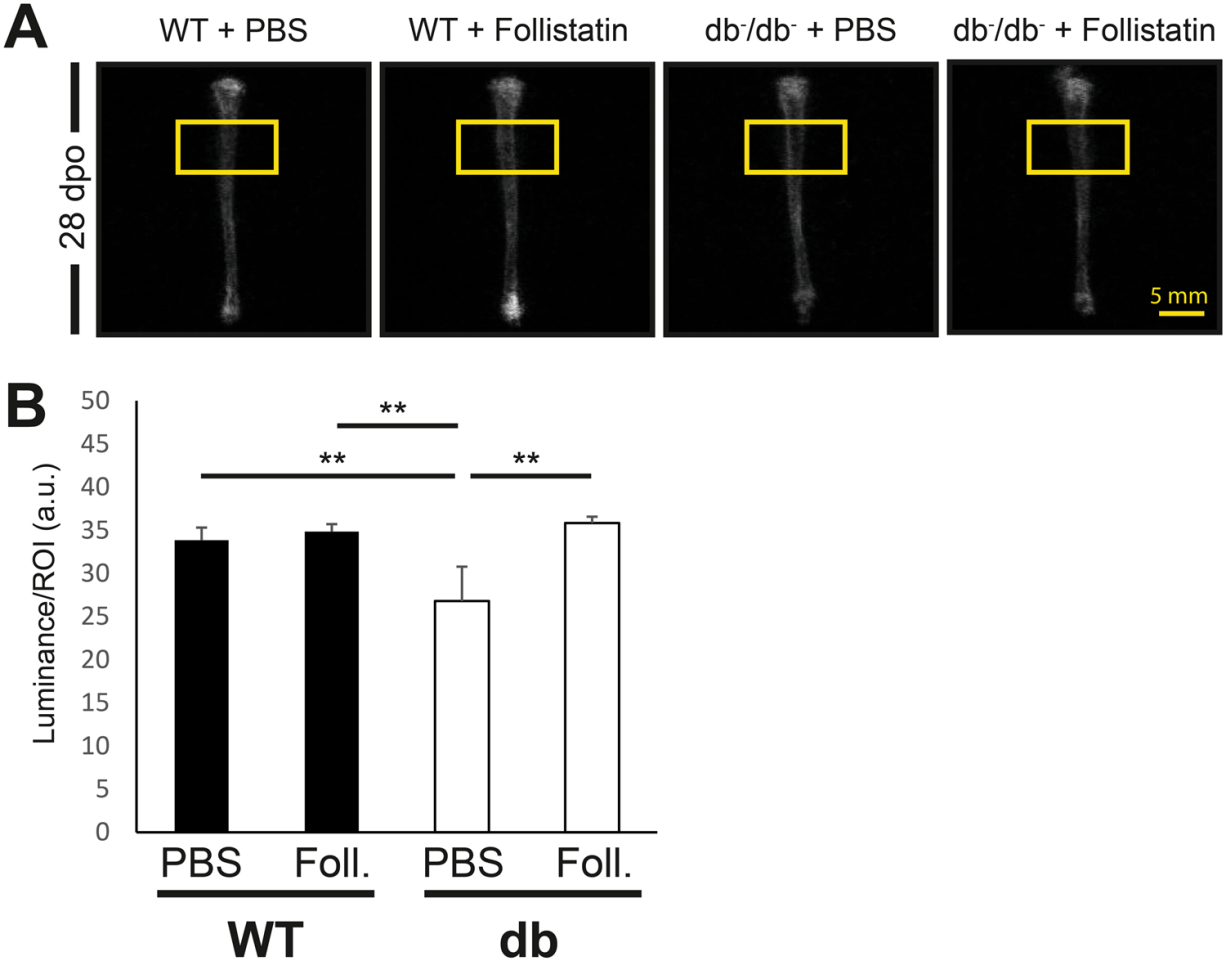
Follistatin enhances diabetic bone regeneration - radiography. **(A)** Representative digital radiography of mouse tibiae 28 days post operatively. Yellow box indicates the site of the defect. **(B)** Measurement of luminance at the defect site in the region of interest (40 × 20 Px) revealed a decrease of bone density in diabetic control tibiae compared to WT tibiae. Administration of Follistatin increased the bone density significantly compared to diabetic control animals. **(B)** Statistical analysis Scale bar: 5 mm. Results are shown as means ± SEM. P-value: * < 0.05; ** < 0.01; (two sample t-test).

With conventional digital radiography of the tibiae 28 days post-operatively bone regeneration was further described. With measuring the luminance of the region of interest (40 × 20 Px). We detected decreased luminance, corresponding with mineral density in diabetic operated tibiae compared to WT operated tibiae (−21%, p = 0.01). Upon single time administration of Follistatin into the tibial defect we demonstrated a restoration of luminance to 105% of the WT level and + 33% compared to the diabetic PBS control defects (p = 0.004) (Fig. 7).

## Discussion

The aim of the study was to investigate the pro-osteogenic effect of Myostatin inhibition by Follistatin in diabetic bone. We could demonstrate that Myostatin is increased in uninjured Lepr^db−/−^ as compared to WT bone in mice. We were able to show no change of Myostatin mRNA expression upon Follistatin treatment. Targeting overexpression of Myostatin in diabetic bone, local application of Follistatin – a potent Myostatin inhibitor – showed a sufficient inhibition of Myostatin and the major downstream targets SMAD 2/3 and p38 in diabetic bone. Concordantly, osteogenic differentiation (RUNX-2), proliferation (PCNA) and osteoid formation were significantly enhanced in diabetic bone upon local application of Follistatin. Moreover, an elevation of PECAM was detected in Follistatin treated bone though not significant.

Although the impact of Myostatin is mostly attributed to muscle tissue, we showed Myostatin activation and subsequently its inhibition upon administration of Follistatin in bone tissue, however there might be an additional impact of Myostatin inhibition through a hypertrophic effect on surrounding muscle. It has been shown that access of bone to muscle is supportive for osseous healing not only by contributing blood supply but also by muscle cells serving as osteoprogenitor cells^39, 40^. Furthermore mechanical forces of muscles may facilitate bone healing^41, 42^. It has been shown that bone mass and strength highly correlates with body weight, whereas lean body mass is particularly associated with bone strength and mineralisation due to mechanical stimuli^43^. Furthermore a novel role of muscle as an endocrine organ is postulated. The secreted cytokines and growth factors from muscle are referred as myokines. From a clinical perspective, fractures covered with a muscle flap heal more quickly^44^. Studies have shown that placing a porous barrier between the muscle coverage and the bone enhances the healing capacity when the pores are large enough for muscle derived factors to diffuse^45^. Myokines such as Interleukin 6 and leukemia inhibitory factor have been shown to stimulate bone formation, while Follistatin has not been determined as a potential pro-osteogenic myokine yet. Hence, there seem to be multiple interaction between muscle and bone upon regeneration, and we just started to understand this reciprocal relationship However Myostatin might be a key factor linking muscle atrophy with bone loss and therefore acts as anti-osteogenic myokine. We were able to show the pro-osteogenic capacity of Follistatin through Myostatin blockade *in vivo* and *in vitro* ^46^.

Mice expressing a dominant-negative Myostatin receptor (activin receptor type IIB) in muscle showed reduced blood glucose, serum insulin, triglyceride levels, and the rate of triglyceride synthesis, and improved insulin sensitivity, while elevated insulin sensitivity was not due to reduced fat mass^11^. Myostatin inhibition re-evaluates the role of muscle metabolism in diabetic disease. The underrated potential of bone and muscle as organs in treating diabetes systemically must be re-evaluated; several glucose transporter types are described to be expressed in different bone cells implicating an important role in glucose-metabolism^47, 48^. Our work emphasizes the underestimated role of Myostatin in bone metabolism and therefore the potential implication in treating systemic diabetic disease.

The role of Follistatin may be central in bone remodelling, as Follistatin-like 1 was demonstrated to be upregulated and secreted during osteoclast differentiation and positively regulates osteoclast formation induced by RANKL^49^. Follistatin is also considered as a partial antagonist of BMPs – however we have demonstrated a similar effect of Follistatin compared to BMPs on bone formation. A common downstream target of BMPs and Follistatin is SMAD 1^20, 22^. BMP-7 for example promotes the expression of RUNX-2 and Osterix directing them in osteoblast differentiation. Accordingly, in our experiments Follistatin significantly enhanced RUNX-2 in diabetic bony defect. Activin A was shown to be involved in positively regulating bone formation, however we were able to show that osteogenic differentiation and SMAD 2/3 decrease were mediated primarily through inhibition of Myostatin by Follistatin. On the other hand it has been shown that Follistatin may interfere with Activin A, consequently inhibiting osteogenic precursor cells to differentiate but enhancing proliferation of osteoblasts^50^. However another study demonstrated an inhibitory effect of Activin A on osteoblast differentiation^16^. Thus, the exact role of Activin A remains elusive and has to be further investigated. GDF 11 as agonist at the Activin receptor 2 was inhibited by Follistatin. Still, no elevation in diabetic bone compared to WT bone was found in our *in vivo* experiments. Interestingly, investigations by Gregorevic and colleagues suggest Follistatin mediated Myostatin independent muscle hypertrophy by SMAD 1/3 and mTOR suggesting a strong involvement of Follistatin on BMP7 activation^22, 26^. However we detected no significant elevation of BMP 7 downstream target SMAD 1 upon Follistatin treatment *in vivo*. Though we found decreased levels of SMAD 1 in diabetic bone compared to WT. Inhibins including Follistatin are important regulators of bone turnover as well and have a bimodal effect on bone metabolism^51^. Inhibin A for example was also demonstrated to stimulate bone formation during distraction osteogenesis^52^. Further experiments are highly demanded to differentiate the strong bimodal role of Follistatin in bone metabolism as partially antagonizing of BMP-2/4 and Activin A. In our study, local application of Follistatin onto injured diabetic bone demonstrated a significant pro-osteogenic and proliferative component of Follistatin. The interaction of Follistatin, Myostatin and recently discovered GDF 11 and BMP 7 is puzzling due to their individual role in bone remodelling and requires separate experimental setups to further characterize their role.

## Conclusion

Our study demonstrated that local inhibition of Myostatin in diabetic bony defects can lead to a significant enhancement of osteogenesis, osteogenic differentiation and proliferation. Our study suggests that potential applications of Follistatin may be expanded and further investigation of Follistatin as local and potential systemic treatment of diabetes and diabetes associated bony defects are warranted.
